# Effects of fermented mulberry leaves on growth performance, nutrient digestibility, diarrhea, and intestinal microecology in weaned piglets: a preliminary study

**DOI:** 10.3389/fvets.2026.1874983

**Published:** 2026-06-26

**Authors:** Rong Wan, Siwei Nong, Chuanfang Zhang, Zhaoxiong Wang, Kaijun Wang, Zhimiao Zou

**Affiliations:** 1 College of Agriculture and Food Engineering, Baise University, Baise, China; 2 College of Animal Science and Technology, Yangtze University, Jingzhou, China; 3 College of Food and Biotechnology, Sichuan Vocational and Technical College, Suining, China; 4 Hunan Provincial Key Laboratory of the Traditional Chinese Medicine Agricultural Biogenomics, Changsha Medical University, Changsha, China

**Keywords:** diarrhea, intestinal microecology, mulberry leaves, nutrient digestibility, piglets

## Abstract

This study investigated the effects of dietary fermented mulberry leaves (FML) on growth metrics, nutrient digestibility, diarrhea incidence, and gut microbial ecology in weaned piglets. A total of 200 piglets (28 days old) were randomly allocated into five groups: a basal diet control group and four treatment groups receiving the basal diet supplemented with 5%, 10%, 15%, or 20% FML (co-fermented with *Lactobacillus* and cellulase) over a 28-day period. Growth performance, apparent nutrient digestibility, diarrhea rate, intestinal pH, short-chain fatty acid (SCFA) concentrations, and cecal microbiota (via 16S rRNA sequencing) were evaluated. No notable differences in growth performance were detected across groups (*P* > 0.05); however, the feed-to-gain ratio was significantly lower in the 5% and 20% FML groups (*P* < 0.05). Diarrhea incidence declined across all FML-supplemented groups, with the greatest reduction (52.46%) in the 15% group during days 28–42. Duodenal pH decreased significantly in all treatment groups (*P* < 0.05). Cecal acetate and propionate levels rose markedly in the 10%, 15%, and 20% groups (*P* < 0.05), with the 15% group showing a 47.05% increase in acetate. FML supplementation also altered cecal microbial diversity and community composition. At the genus level, the 15% group had the highest relative abundance of *Prevotella* (22.46%), while *Lactobacillus* and *Bifidobacterium* tended to increase in FML groups. Overall, dietary inclusion of 5–20% FML did not significantly enhance growth performance but effectively reduced diarrhea, optimized intestinal pH, increased cecal acetate and propionate production, and modulated cecal microbiota composition, with 15% FML identified as the optimal level.

## 1 Introduction

Weaning represents a critical phase in modern swine production, during which piglets frequently experience stress due to dietary and environmental transitions. This stress often manifests as villus atrophy, impaired nutrient absorption, diarrhea, and subsequent growth suppression (1, 2). The intestinal barrier comprises physical, chemical, immunological, and microbial components (3), serving as a vital defense against external harmful agents. Gut microorganisms and their metabolic byproducts contribute to intestinal protection by reinforcing barrier function, resisting pathogen colonization, reducing inflammation, and preserving homeostasis (4, 5). Therefore, supporting intestinal barrier integrity and maintaining balanced gut microbiota and their metabolites are essential strategies for promoting intestinal health in weaned piglets.

Natural medicinal plants contain a wide range of active constituents that can enhance digestive secretion, maintain intestinal barrier integrity, and exert antibacterial, anti-inflammatory, and antioxidant effects (6–9). Mulberry leaves and twigs are rich in crude protein, essential amino acids, trace minerals (10), and various bioactive compounds such as polyphenols, polysaccharides, and alkaloids. These components exhibit antioxidant, immunomodulatory, anti-stress, and lipid-lowering properties (11), positioning mulberry leaves as a promising protein feed source for livestock (12). However, fresh mulberry leaves contain high levels of lignified fiber and antinutritional factors like tannins and wax (13), limiting their direct use as feed. Fermentation has been shown to convert plant components into proteins and small peptides, reduce antinutritional factors, release flavonoids (14), and improve the nutritional profile of plant-based feeds (15, 16), while also enhancing gut morphology and microbial balance (17, 18).

Previous studies have indicated that mulberry leaf powder can serve as a protein source for dairy cows (19) and sheep (20), with digestible energy and crude protein levels comparable to alfalfa hay (21, 22). In weaned piglets, supplementation with 6% mulberry leaf powder negatively impacted growth performance (23), while 9% inclusion in finishing pigs increased Bifidobacterium abundance (24). FML has also been shown to reshape intestinal microbial balance in finishing pigs (25). Nonetheless, due to the high fiber and antinutritional factor content in mulberry leaves, direct inclusion yields inconsistent results and is constrained by inclusion thresholds.

Microbial fermentation and enzymatic hydrolysis are increasingly recognized as innovative feed processing techniques in animal production (26). Fermenting mulberry leaves with lactic acid bacteria elevates acetic acid content, reduces undesirable microbes such as yeasts, molds, and Escherichia coli, and enhances antioxidant activity along with the abundance of Lactococcus and Lactobacillus (27), while lowering anti-nutritional factors (28). Adding cellulase during fermentation facilitates fiber breakdown into soluble carbohydrates, providing substrates for lactic acid bacteria and sustaining acid production (29). Advances in sequencing enable deeper studies on how diet affects animal gut microbiota (30–33). Despite growing interest in FML as a feed ingredient, research on its application in weaned piglets—especially when co-fermented with both lactic acid bacteria and cellulase—remains limited. Moreover, systematic evaluations of its effects on intestinal microecology using high-throughput sequencing are scarce. Accordingly, this study employed FML as the experimental material to assess growth performance, diarrhea incidence, intestinal pH, SCFA levels, and cecal microbiota via 16S rRNA sequencing, aiming to elucidate the mechanisms by which FML influences intestinal health in weaned piglets and to provide a theoretical basis for its use in production.

## 2 Materials and methods

All procedures were approved by the Animal Care and Use Committee of Baise University (protocol number: BSUCKL20260003).

### 2.1 Experimental design, diets and management

A total of 200 weaned piglets (Duroc × Landrace × Yorkshire, 28 days old, equal numbers of castrated males and females) with similar initial body weight and age were randomly assigned to five treatment groups. Each group consisted of four replicates with ten piglets per replicate. Following a 2-day adaptation period (days 28–30 of age), the formal trial lasted 28 days (days 30–58 of age). A completely randomized single-factor design was implemented, with FML inclusion levels of 0% (control, CK), 5%, 10%, 15%, and 20% in the basal diet.

The basal corn–soybean meal diet was formulated to meet the nutritional requirements of 5–10 kg piglets as recommended by NRC (2012). Diets containing varying FML levels were adjusted to maintain consistent digestible energy and crude protein content across all groups. The ingredient composition and nutritional profiles are presented in Table 1.

**Table 1:** Ingredient composition and nutrient levels of the diets (dry matter basis).

Ingredients	CK	5% FML	10% FML	15% FML	20% FML
Corn	32.23	29.70	20.68	19.03	17.00
Corn extruded	20.00	20.00	20.00	20.00	20.00
Soybean meal	7.59	0	0	0	0
Extruded full-fat soybean	6.67	10.00	15.00	15.00	10.96
Soy protein	8.00	9.73	8.82	5.47	6.54
Dried whey	10.00	10.00	10.00	10.00	10.00
Chicken liver meal	3.00	3.00	3.00	3.00	3.00
Yeast hydrolysate	3.00	3.00	3.00	3.00	3.00
Soybean oil	1.50	1.50	1.50	1.50	1.50
Premix^1^	8.00	8.00	8.00	8.00	8.00
Fermented mulberry leaves	0	5	10	15	20
Total	100.00	100.00	100.00	100.00	100.00
Nutrient levels					
DE, Kcal/kg	3,400	3,400	3,400	3,400	3,400
CP, %	18.20	18.27	18.32	18.34	18.23
Lys, %	1.45	1.45	1.45	1.45	1.45
Met, %	0.60	0.60	0.60	0.60	0.60
Met+Cys, %	0.88	0.88	0.88	0.88	0.88
Thr, %	0.96	0.96	0.96	0.96	0.96

^1^Premix provided per kilogram of diet: 11,000 IU retinol; 1,500 IU cholecalciferol; 44 IU α-tocopherol; 4 mg menadione; 2.5 mg thiamine; 5.2 mg riboflavin; 26 mg nicotinic acid; 20 mg pantothenic acid; 4 mg pyridoxine; 0.02 mg cyanocobalamin; 4 mg biotin; 50 mg folic acid; 35 mg Mn; 90 mg Zn; 100 mg Fe; 16.5 mg Cu; 0.3 mg I; 0.3 mg Se.

The trial was conducted at a commercial pig farm (Guangxi Pig Pastoral Ecological Feed Co., Ltd.). Piglets were housed in elevated nursery pens under controlled conditions (25–28°C, 55–65% relative humidity). Feed and water were provided *ad libitum*, and feeding schedules followed farm protocols. Individual body weights were recorded after an overnight fast on days 28, 42, and 56 of age (i.e., days 1, 14, and 28 of the formal trial). Routine management practices were applied uniformly across all groups.

### 2.2 Preparation of FML

Fresh mulberry leaves were harvested, crushed and co-fermented with *Lactobacillus plantarum* (CICC 10146, viable count ≥ 1 × 10^11^ CFU/g) at 10^8^ CFU/g and cellulase (enzyme activity ≥ 10 000 U/g) at 500 mg/kg for 60 days. Both microorganisms and enzyme were sourced from Shandong Weilan Biotechnology Co., Ltd. Fermentation was carried out at 30 ± 2°Cwith an initial humidity of 65% and initial pH of 5.8. The fermentation endpoint was determined when the pH remained stable between 4.2 and 4.5 for three consecutive days and the lactic acid bacteria count exceeded 1 × 10^10^ CFU/g.

The nutrient composition of the resulting FML was as follows: dry matter (DM) 47.70%, crude protein (CP) 14.40%, ether extract (EE) 2.70%, crude fiber (CF) 14.73%, ash 8.90%, calcium (Ca) 2.59% and total phosphorus (TP) 0.62%. Lactic acid bacteria count reached 1.05 × 10^11^ CFU/g, with no detectable molds.

### 2.3 Growth performance

Individual body weights were recorded after a 16-h fast on days 28, 42 and 56 of age. Average daily feed intake (ADFI), average daily gain (ADG) and feed-to-gain ratio (F/G) were calculated for each replicate.

### 2.4 Nutrient apparent digestiblity

Fresh fecal samples (approximately 200 g) were collected from each replicate on days 54–56 of age (i.e., days 26–28 of the formal trial). Samples were divided into two portions: one was treated with 10% sulfuric acid for nitrogen fixation to determine CP, and the other was used for other nutrient analyses. All samples were dried at 65°C to constant weight, equilibrated to room temperature for 24 h, ground through a 40-mesh sieve and stored. Diet samples were processed similarly. Apparent digestibility was determined using acid-insoluble ash (AIA) as an indigestible marker. DM, CP, EE, CF, ash, Ca and TP contents in both diets and feces were analyzed following Chuanfang et al. (34). AIA content was measured using the 4 mol/L HCl-insoluble ash method. Digestibility was calculated as follows: Apparent digestibility (%) = [1 – (A_1_/A_2_) × (N_2_/N_1_)] × 100, where A_1_ and A_2_ represent AIA concentrations in the diet and feces, respectively, and N_1_ and N_2_ are the nutrient concentrations in the diet and feces, respectively.

### 2.5 Diarrhea incidence

Fecal consistency was monitored and scored daily after morning feeding. Diarrhea incidence (%) was calculated as [total diarrhea cases / (total piglets × trial days)] × 100%. Scoring criteria: 0 = normal (formed pellets or strips), 1 = mild diarrhea (soft but formable), 2 = moderate diarrhea (pasty, unformed), 3 = severe diarrhea (liquid, separated).

### 2.6 Short-chain fatty acid concentration and pH in intestinal digesta

On day 56 of age, one piglet per replicate was selected and humanely euthanized according to the national standard GB/T 39760-2021 (35). Animals were sedated with ketamine (20 mg/kg) and xylazine (2 mg/kg) intramuscularly, followed by intravenous sodium pentobarbital (120 mg/kg) via the auricular vein. Exsanguination was performed to confirm death. Tissue collection began only after cessation of vital signs. Segments (approximately 3 cm) of the mid-jejunum and cecum were collected, ligated, wrapped in foil and plastic wrap, and stored in liquid nitrogen for microbial analysis. Cecal digesta were collected for SCFA determination.

Intestinal digesta pH was measured immediately postmortem in the duodenum, jejunum, ileum, cecum, and colon using a pH90 direct-reading pH meter.

For SCFA analysis, approximately 1.0 g of cecal digesta was mixed 1:1 with distilled water, vortexed, and centrifuged at 4,000 rpm for 10 min. One milliliter of supernatant was further centrifuged at 12,000 rpm for 10 min. Then, 900 μL of supernatant was mixed with 0.2 mL of 25% metaphosphoric acid, incubated at 4°C for 30 min, and centrifuged again. A 200 μL aliquot was mixed with 200 μL methanol, centrifuged, and the supernatant stored at −20°C for gas chromatography analysis.

For microbial enumeration, 0.5 g of intestinal content was mixed with 49.5 mL sterile nutrient broth, serially diluted (10^−2^ to 10^−10^), and inoculated onto selective media. *Escherichia coli* was cultured on MacConkey agar (aerobic, 37°C, 24 h), *Lactobacillus* on MRS agar (anaerobic, 37°C, 48 h), and *Bifidobacterium* on BBL agar (anaerobic, 37°C, 48 h). Colony identification was based on typical morphology, Gram staining, and catalase reaction. All media were purchased from Qingdao Hope Bio-Technology Co., Ltd. The jejunum was chosen for enumeration because it is a key site for nutrient absorption and host-microbe interactions in weaned piglets.

### 2.7 16S rRNA sequencing and cecal microbiota analysis

Cecal digesta from three piglets per group (selected as those closest to the average body weight) were collected aseptically and stored at −80°C. Total DNA was extracted using the E.Z.N.A.^®^ Soil DNA Kit (Omega Bio-tek), and DNA quality was assessed via NanoDrop ND-2000 (Thermo Fisher Scientific). The V3–V4 region of the 16S rRNA gene was amplified using primers 338F and 806R. Purified amplicons were sequenced on an Illumina Nextseq2000 platform (Shanghai Majorbio Bio-pharm Technology Co., Ltd.). Raw data were processed using the DADA2 pipeline in QIIME2 (2020.2) to generate amplicon sequence variants (ASVs) and an abundance table. Taxonomic annotation, alpha diversity, and beta diversity analyses were performed based on ASVs (36).

### 2.8 Statistical analysis

Data were analyzed using SAS version 9.0 (SAS Institute Inc.). Normality and homogeneity of variance were verified. One-way ANOVA followed by Duncan's multiple range test was used to compare treatment groups, with each replicate considered an experimental unit. Results are expressed as means ± SEM. Significance was declared at *P* < 0.05 (significant) or *P* < 0.01 (highly significant). Alpha diversity indices were computed using mothur, and group differences were assessed via Wilcoxon rank-sum test. Beta diversity was visualized using principal coordinate analysis (PCoA) based on Bray-Curtis distances, with statistical significance tested by PERMANOVA. Linear discriminant analysis effect size (LEfSe) was applied to identify differentially abundant taxa (LDA score > 2, *P* < 0.05).

## 3 Results

### 3.1 Effects of FML on growth performance

As shown in Table 2, no significant differences in ADFI, ADG, or F/G were observed between any FML group and the control during days 28–42 (*P* > 0.05). From days 42–56, ADG was numerically higher in all FML groups compared with the control, with the 20% group showing a 14.78% increase, though not statistically significant (*P* = 0.134). F/G differed significantly among groups (*P* = 0.047), with the 5% and 20% groups showing lower F/G than the control (*P* < 0.05). Over the full trial period (days 28–56 of age), ADFI and ADG were slightly elevated and F/G slightly reduced in FML groups compared with the control, but differences were not significant (*P* > 0.05). These findings suggest that dietary FML at 5–20% does not significantly affect overall growth performance, though 20% supplementation tended to improve ADG and F/G during the later phase.

**Table 2:** Effects of fermented mulberry leaves on growth performance of weaned piglets.

Item	CK	5% FML	10% FML	15% FML	20% FML	*P*-Value
Days 28~42						
ADFI, g	447.37 ± 61.10	442.68 ± 78.13	444.64 ± 40.76	443.58 ± 31.04	439.26 ± 41.08	0.863
ADG, g	235.35 ± 31.38	251.34 ± 12.61	237.96 ± 22.92	238.84 ± 32.89	234.93 ± 10.68	0.748
F/G	1.90 ± 0.21	1.76 ± 0.23	1.87 ± 0.18	1.86 ± 0.19	1.87 ± 0.23	0.243
Days 42~56						
ADFI, g	469.66 ± 37.93	483.12 ± 40.09	506.92 ± 49.28	490.98 ± 14.86	498.00 ± 17.15	0.345
ADG, g	234.38 ± 9.48	264.84 ± 9.17	255.72 ± 23.55	249.28 ± 25.91	269.02 ± 22.80	0.134
F/G	2.00 ± 0.25^a^	1.82 ± 0.16^b^	1.98 ± 0.19^ab^	1.97 ± 0.22^ab^	1.85 ± 0.19^b^	0.047
Days 28~56						
ADFI, g	459.02 ± 25.71	463.40 ± 41.49	476.28 ± 29.04	467.98 ± 15.02	469.13 ± 21.86	0.612
ADG, g	234.87 ± 11.77	258.09 ± 7.39	246.84 ± 14.81	244.06 ± 14.64	251.98 ± 8.04	0.366
F/G	1.95 ± 0.21	1.80 ± 0.15	1.93 ± 0.17	1.92 ± 0.20	1.86 ± 0.16	0.395

F/G, feed-to-gain ratio; ADFI, average daily feed intake; ADG, average daily gain. ^a, b^Means within the same row that have no common superscript are significantly different (*P* < 0.05). CK, control group; 5%FML, group receiving a diet with 5% FML; 10%FML, group receiving a diet with 10% FML; 15%FML, group receiving a diet with 15% FML; 20%FML, group receiving a diet with 20% FML. FML, fermented mulberry leaves.

### 3.2 Effects of FML on nutrient apparent digestibility

Table 3 presents the effects on nutrient digestibility. As FML inclusion increased, apparent digestibility of DM, CP, and CF gradually declined. DM digestibility was significantly lower in the 10%, 15%, and 20% groups compared with the control (*P* < 0.05), with reductions of 1.88%, 2.32%, and 3.15%, respectively. CP digestibility was significantly reduced in the 10% and 15% groups relative to the control and 5% groups (*P* < 0.05), with the 20% group showing the lowest value (6.96% reduction). CF digestibility was highest in the control group but the differences were not statistically significant (*P* = 0.090). In contrast, EE digestibility increased with FML inclusion, with the 15% and 20% groups showing significantly higher values than the control, 5%, and 10% groups (*P* < 0.05), representing increases of 5.13% and 5.31%, respectively. No significant differences were observed among groups for ash, Ca, or TP digestibility (*P* > 0.05).

**Table 3:** Effects of fermented mulberry leaves on nutrient apparent digestibility in weaned piglets.

Item	CK	5% FML	10% FML	15% FML	20% FML	*P*-Value
DM, %	80.94 ± 0.62^a^	80.90 ± 1.24^a^	79.42 ± 0.22^b^	79.06 ± 0.43^b^	78.39 ± 0.26^b^	0.003
CP, %	77.49 ± 1.09^a^	76.76 ± 0.81^a^	74.27 ± 1.06^b^	74.04 ± 1.06^b^	72.10 ± 0.35^c^	0.000
EE, %	64.09 ± 1.10^b^	64.75 ± 0.94^b^	65.29 ± 1.07^b^	67.38 ± 0.95^a^	67.49 ± 1.07^a^	0.006
CF, %	43.27 ± 0.94	42.23 ± 0.94a	41.25 ± 0.96	41.08 ± 0.34	40.53 ± 0.18	0.090
Ash, %	42.67 ± 0.33	41.97 ± 0.62	42.54 ± 0.17	42.10 ± 0.66	41.55 ± 1.46	0.460
Ca, %	52.01 ± 0.56	51.32 ± 1.02	51.29 ± 1.00	51.47 ± 1.16	51.67 ± 0.57	0.857
TP, %	74.52 ± 0.85	73.10 ± 1.53	73.77 ± 0.30	72.66 ± 0.32	72.62 ± 0.95	0.103

DM, dry matter; CP, crude protein; EE, ether extract; CF, crude fiber; Ca, calcium; TP, total phosphorus. ^a, b^Means within the same row that have no common superscript are significantly different (*P* < 0.05). CK, control group; 5%FML, group receiving a diet with 5% FML; 10%FML, group receiving a diet with 10% FML; 15%FML, group receiving a diet with 15% FML; 20%FML, group receiving a diet with 20% FML. FML, fermented mulberry leaves.

### 3.3 Effects of FML on diarrhea incidence

During days 28–42 of age, diarrhea incidence was significantly lower in all FML groups than in the control (*P* < 0.05), with the 15% group showing the greatest reduction (52.46%), followed by 39.34%, 18.03%, and 34.43% reductions in the 5%, 10%, and 20% groups, respectively. No significant differences were observed among groups during days 42–56 (*P* > 0.05). Over the entire trial period (days 28–56), the 15% group maintained a significantly lower diarrhea rate compared with the control (*P* < 0.05), corresponding to a 41.86% reduction (Table 4). These results indicate that FML supplementation effectively reduces diarrhea, with 15% being the most effective level.

**Table 4:** Effects of fermented mulberry leaves on diarrhea incidence in weaned piglets.

Item	CK	5% FML	10% FML	15% FML	20% FML	*P*-Value
Days 28~42						
Diarrhea rate, %	0.61 ± 0.07^a^	0.37 ± 0.04^ab^	0.50 ± 0.04^ab^	0.29 ± 0.03^b^	0.40 ± 0.03^ab^	0.046
Days 42~56						
Diarrhea rate, %	0.24 ± 0.02	0.27 ± 0.03	0.23 ± 0.01	0.21 ± 0.02	0.26 ± 0.02	0.586
Days 28~56						
Diarrhea rate, %	0.43 ± 0.04^a^	0.32 ± 0.03^ab^	0.37 ± 0.02^ab^	0.25 ± 0.03^b^	0.33 ± 0.04^ab^	0.050

^a, b^Means within the same row that have no common superscript are significantly different (*P* < 0.05). CK, control group; 5%FML, group receiving a diet with 5% FML; 10%FML, group receiving a diet with 10% FML; 15%FML, group receiving a diet with 15% FML; 20%FML, group receiving a diet with 20% FML. FML, fermented mulberry leaves.

### 3.4 Effects of FML on intestinal pH and SCFAs

As shown in Table 5, duodenal pH was significantly lower in all FML groups compared with the control (*P* < 0.05), with reductions of 6.92%, 3.91%, 7.07% and 7.97% in the 5%, 10%, 15% and 20% groups, respectively. No significant differences were observed in jejunal, ileal, cecal or colonic pH among groups (*P* > 0.05). Ileal pH tended to decrease numerically (from 6.85 in CK to 6.50 in the 20% group, *P* = 0.133), and cecal pH showed a similar trend (from 6.18 to 5.91, *P* = 0.053).

**Table 5:** Effects of fermented mulberry leaves on short-chain fatty acids concentration and pH value in intestinal digesta of weaned piglets.

Item	CK	5% FML	10% FML	15% FML	20% FML	*P*-Value
pH value in intestinal digesta						
Duodenum	6.35 ± 0.52^a^	5.89 ± 0.45^b^	6.09 ± 0.53^ab^	5.82 ± 0.40^b^	5.88 ± 0.47^b^	0.040
Jejunum	6.70 ± 0.45	5.83 ± 0.33	6.67 ± 0.46	6.55 ± 0.40	6.41 ± 0.58	0.150
Ileum	6.85 ± 0.35	6.81 ± 0.37	6.66 ± 0.63	6.73 ± 0.25	6.50 ± 0.57	0.133
Cecum	6.18 ± 0.40	6.16 ± 0.28	6.03 ± 0.42	5.95 ± 0.23	5.91 ± 0.29	0.053
Colon	6.07 ± 0.48	6.13 ± 0.35	6.01 ± 0.25	6.12 ± 0.50	5.83 ± 0.52	0.324
SCFAs in the cecum						
Acetate, mmol/L	24.61 ± 3.10^b^	26.08 ± 3.25^b^	34.32 ± 3.22^a^	34.38 ± 5.33^a^	36.33 ± 5.33^a^	0.000
Propionate, mmol/L	15.12 ± 2.39^b^	13.76 ± 3.10^b^	24.27 ± 3.28^a^	22.07 ± 2.06^a^	22.83 ± 3.01^a^	0.001
Butyrate, mmol/L	1.97 ± 0.27	1.22 ± 0.33	1.63 ± 0.62	1.83 ± 0.74	1.71 ± 0.33	0.891
Bacterial count in the jejunum						
Total bacterial count, cfu/g	3.63 ± 0.40^c^	4.51 ± 0.24^ab^	4.09 ± 0.43^abc^	4.30 ± 0.13^ab^	4.60 ± 0.07^a^	0.008
Escherichia coli, cfu/g	3.83 ± 0.40^b^	4.01 ± 0.38^b^	4.09 ± 0.51^b^	5.37 ± 0.69^a^	4.16 ± 0.38^b^	0.105
Lactobacillus, cfu/g	3.62 ± 0.56^b^	4.69 ± 0.44^a^	4.26 ± 0.18^ab^	4.20 ± 0.77^ab^	4.15 ± 0.52^ab^	0.565
Bifidobacterium, cfu/g	3.82 ± 0.46^b^	4.86 ± 0.59^a^	4.38 ± 0.37^ab^	4.54 ± 0.55^ab^	4.12 ± 0.54^ab^	0.796
Bacterial count in the cecum						
Total bacterial count, cfu/g	6.20 ± 0.73^abc^	6.78 ± 0.69^ab^	5.65 ± 0.77^c^	6.50 ± 0.38^ab^	6.85 ± 0.68^a^	0.293
Escherichia coli, cfu/g	5.70 ± 0.92^ab^	6.23 ± 0.50^a^	4.89 ± 0.37^b^	6.19 ± 0.53^a^	6.55 ± 0.67^a^	0.154
Lactobacillus, cfu/g	6.48 ± 0.57	6.95 ± 0.63	6.98 ± 0.29	6.93 ± 0.50	6.84 ± 0.82	0.424
Bifidobacterium, cfu/g	6.92 ± 0.87	7.39 ± 0.79	6.83 ± 0.70	6.86 ± 0.19	7.29 ± 0.74	0.828

^a, b, c^Means within the same row that have no common superscript are significantly different (*P* < 0.05). CK, control group; 5%FML, group receiving a diet with 5% FML; 10%FML, group receiving a diet with 10% FML; 15%FML, group receiving a diet with 15% FML; 20%FML, group receiving a diet with 20% FML. FML, fermented mulberry leaves.

Cecal acetate concentrations in the 10%, 15%, and 20% groups were significantly higher than in the control and 5% groups (*P* < 0.01), with increases of 38.98%, 47.05%, and 39.22%, respectively. Propionate levels were also significantly elevated in these groups, with increases of 59.33%, 45.07%, and 50.00% compared with the control (*P* < 0.05). No significant differences were observed in butyrate levels across groups (*P* > 0.05).

Jejunal microbial enumeration revealed that total bacterial counts were significantly lower in the control group than in the 5%, 15%, and 20% groups (*P* < 0.01). *Lactobacillus* and *Bifidobacterium* counts were significantly higher in the 5% group than in the control (*P* < 0.05), with increasing trends in other FML groups. Cecal enumeration showed no significant differences in total bacteria, *Escherichia coli, Lactobacillus*, or *Bifidobacterium* counts (*P* > 0.05), though *Lactobacillus* counts were numerically higher in all FML groups.

### 3.5 Effects of FML on cecal microbiota

**Figure 1:**
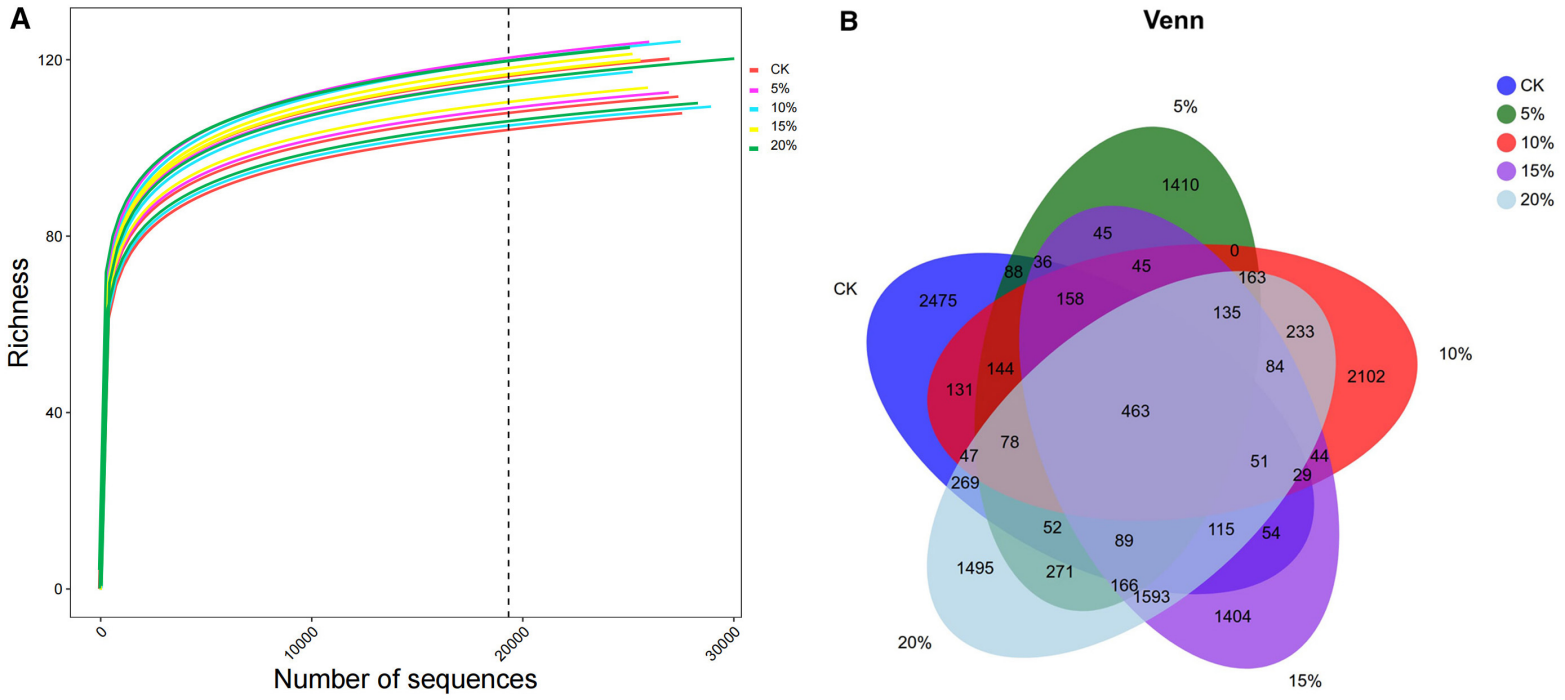
**(A, B)** Quality analysis of 16S rRNA sequencing. **(A)** Dilution curve. The dilution curve is used to indicate whether the sequencing data volume of the sample is sufficient. When the curve tends to flatten toward the end, it indicates that the sequencing data volume is reasonable. The horizontal axis represents the volume of randomly selected sequencing data (number of reads sampled); the vertical axis represents the number of observed species or the Alpha diversity index (Shannon index). **(B)** Venn diagram. Venn diagram is used to display intersections and unions either within or between groups, aiding in the analysis and comparison of similarities and differences among data. CK, control group; 5%FML, group receiving a diet with 5% FML; 10%FML, group receiving a diet with 10% FML; 15%FML, group receiving a diet with 15% FML; 20%FML, group receiving a diet with 20% FML. FML, fermented mulberry leaves.

Dilution curves plateaued (Figure 1A), indicating adequate sequencing depth. The Venn diagram (Figure 1B) showed total ASV counts of 4,279 (control), 3,343 (5%), 3,907 (10%), 4,511 (15%), and 5,301 (20%), with 463 shared ASVs across all groups.

Alpha diversity analysis (Table 6) revealed no significant differences in Observed species, ACE, Shannon, or Pielou indices (*P* > 0.05). The Chao1 index was significantly higher in the 15% and 20% groups than in the control (*P* < 0.05). The Simpson index was highest in the control group and significantly higher than in the 5%, 10%, and 20% groups (*P* < 0.05), indicating reduced bacterial dominance and increased evenness in FML groups. The PD index was significantly higher in the 5% and 10% groups than in the control (*P* < 0.05), suggesting increased phylogenetic diversity.

**Table 6:** Alpha diversity analysis of cecal microbiota in weaned piglets.

Item	CK	5% FML	10% FML	15% FML	20% FML	*P*-Value
Obs	104.00 ± 2.65	113.00 ± 7.21	114.33 ± 7.02	105.66 ± 8.38	110.66 ± 7.58	0.064
Chao1	104.11 ± 2.71^a^	107.07 ± 6.98^ab^	111.58 ± 4.76^ab^	113.17 ± 1.49^b^	114.80 ± 2.29^b^	0.045
ACE	104.22 ± 2.61	113.33 ± 7.32	114.91 ± 7.18	106.12 ± 8.41	111.90 ± 1.48	0.071
Shannon	6.64 ± 0.28	6.09 ± 0.37	6.10 ± 0.36	6.24 ± 0.18	6.46 ± 0.21	0.081
Simpson	0.98 ± 0.01^a^	0.91 ± 0.45^b^	0.92 ± 0.02^b^	0.93 ± 0.01^a^	0.92 ± 0.01^b^	0.036
Pielou	0.64 ± 0.09	0.65 ± 0.08	0.65 ± 0.07	0.69 ± 0.03	0.68 ± 0.04	0.083
PD	12.86 ± 1.56^b^	15.41 ± 0.73^a^	15.23 ± 0.56^a^	14.04 ± 0.94^ab^	14.27 ± 1.03^ab^	0.031

Obs, Observed species; Chao1, Chao estimator; ACE, Abundance-based Coverage Estimator; Shannon, Shannon-Wiener index; Simpson, Simpson diversity index; Pielou, Pielou's Evenness; PD, Phylogenetic Diversity. ^a, b^Means within the same row that have no common superscript are significantly different (*P* < 0.05). CK, control group; 5%FML, group receiving a diet with 5% FML; 10%FML, group receiving a diet with 10% FML; 15%FML, group receiving a diet with 15% FML; 20%FML, group receiving a diet with 20% FML. FML, fermented mulberry leaves.

**Figure 2:**
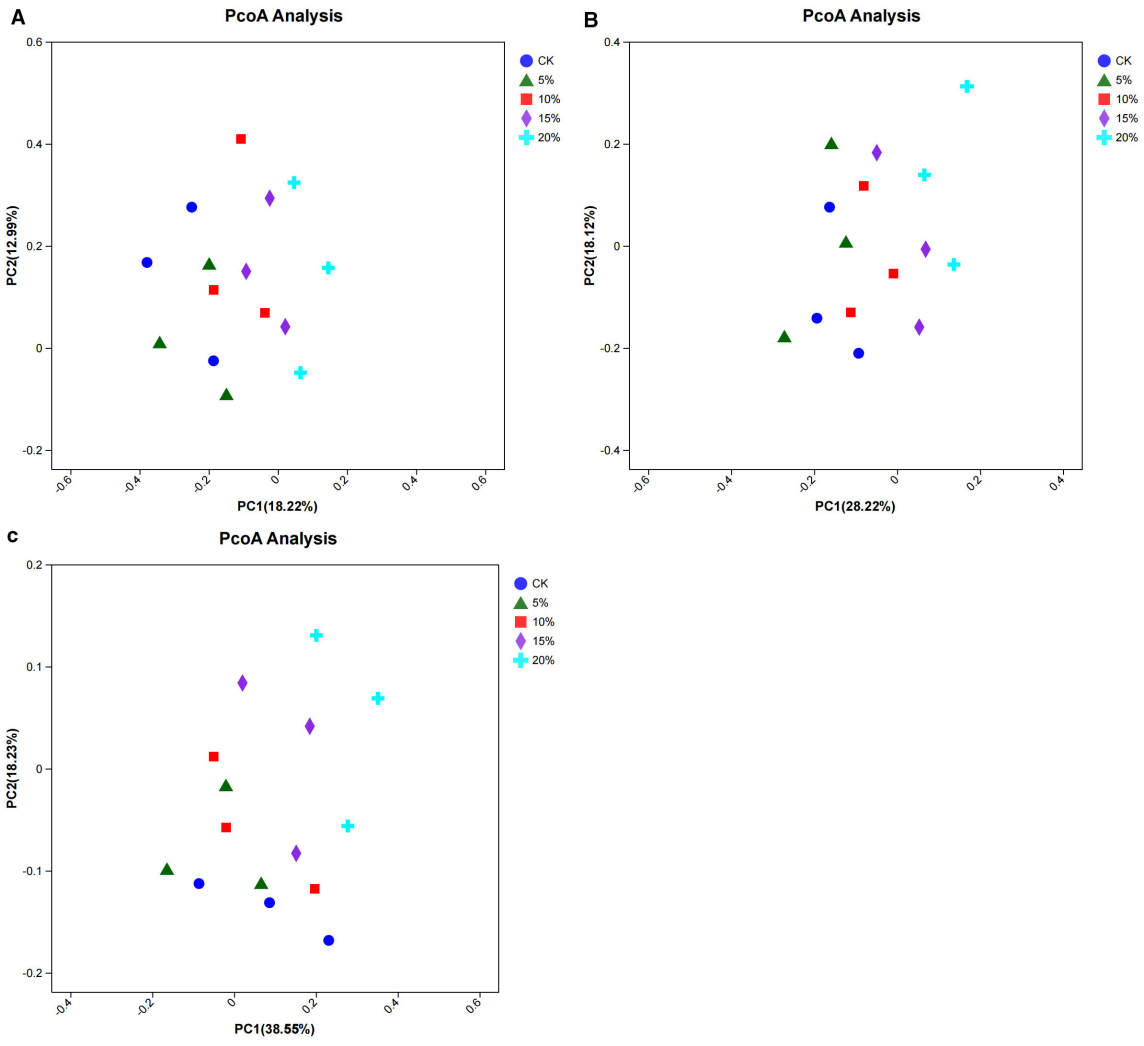
**(A, B, C)** Principal coordinate analysis of PCoA in the cecal microbial community of weaned piglets. **(A)** PCoA analysis based on Bray–Curtis distance; **(B)** PCoA based on unweighted UniFrac distance; **(C)** PCoA based on weighted UniFrac distance. The horizontal and vertical axes correspond to the two selected principal coordinates (PC1 and PC2), with the percentages indicating the proportion of total variance in community composition explained by each coordinate. The axis scales represent unitless scores and are not directly interpretable as absolute distances. Data points are distinguished by color or shape according to their experimental group. The proximity between any two points reflects the similarity in their species composition. CK, control group; 5%FML, group receiving a diet with 5% FML; 10%FML, group receiving a diet with 10% FML; 15%FML, group receiving a diet with 15% FML; 20%FML, group receiving a diet with 20% FML. FML, fermented mulberry leaves.

PCoA based on Bray–Curtis, unweighted UniFrac, and weighted UniFrac distances (Figures 2A–C) showed clear separation between the control and the 15% and 20% groups, indicating FML-induced shifts in cecal microbial structure.

**Figure 3:**
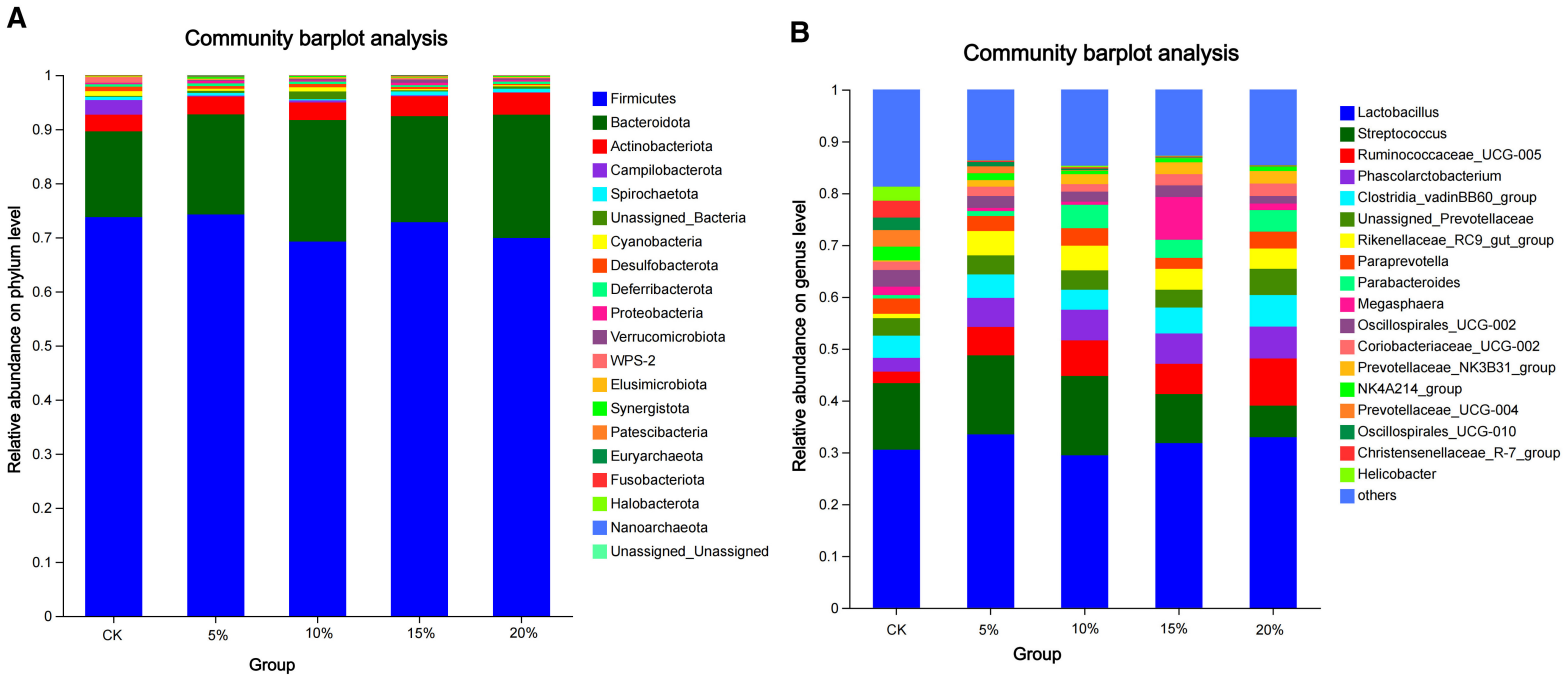
**(A, B)** Microbial community analysis at phylum level, genus level. **(A)** Microbial community analysis at phylum level; **(B)** Microbial community analysis at genus level. CK, control group; 5%FML, group receiving a diet with 5% FML; 10%FML, group receiving a diet with 10% FML; 15%FML, group receiving a diet with 15% FML; 20%FML, group receiving a diet with 20% FML. FML, fermented mulberry leaves.

At the phylum level (Figure 3A), Firmicutes and Bacteroidetes dominated across groups, accounting for > 90% of relative abundance. At the genus level (Figure 3B), *Prevotella* was the most abundant genus, with the highest relative abundance in the 15% group (22.46%). *Lactobacillus* and *Bifidobacterium* abundances were generally higher in FML groups.

**Figure 4:**
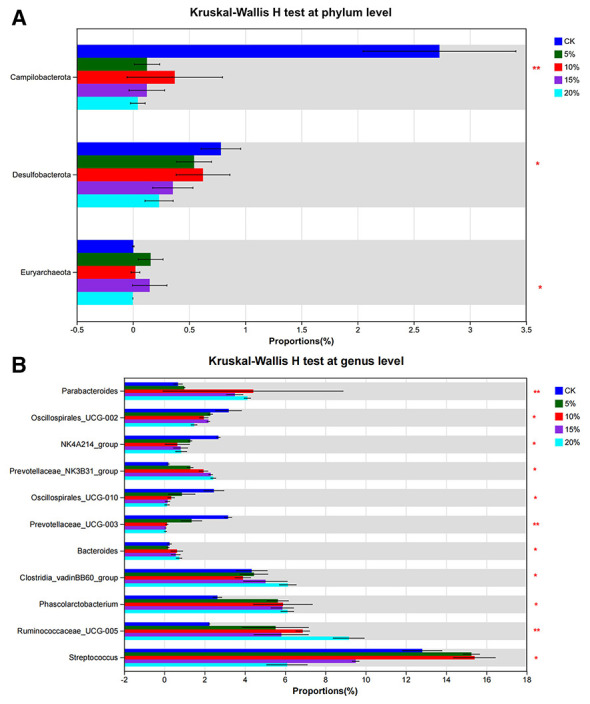
**(A, B)** Kruskal-Wallis rank sum test among groups at phylum level, genus level. **(A)** Kruskal-Wallis rank sum test among groups at phylum level; **(B)** Kruskal-Wallis rank sum test among groups at genus level. CK, control group; 5%FML, group receiving a diet with 5% FML; 10%FML, group receiving a diet with 10% FML; 15%FML, group receiving a diet with 15% FML; 20%FML, group receiving a diet with 20% FML. FML, fermented mulberry leaves. ^*^ indicates *P* < 0.05. ^**^indicates *P* < 0.01.

Kruskal–Wallis tests (Figure 4) showed no significant differences at the phylum level (*P* > 0.05). At the genus level, seven genera differed significantly among groups (*P* < 0.05). *Prevotella* was most abundant in the 15% group, significantly higher than in the control and 5% groups. *Lactobacillus* was most abundant in the 10% group, significantly higher than in the control. *Bifidobacterium* was most abundant in the 15% group, significantly higher than in the control and 20% groups. *Lachnospiraceae_NK4A136_group* was most abundant in the 20% group, significantly higher than in the control.

## 4 Discussion

Weaning stress frequently impairs intestinal integrity, reduces nutrient absorption, and induces diarrhea in piglets, thereby hindering growth (37). In this study, no significant differences in growth performance were observed between FML-supplemented groups and the control over the entire experimental period, indicating that 5–20% FML does not adversely affect growth. Notably, the 20% group exhibited a 14.78% increase in ADG during days 42–56 (*P* = 0.134, not significant), and both the 5% and 20% groups showed significantly reduced F/G during this phase, suggesting that higher FML levels may benefit later-stage growth. Peng et al. (38) reported that fermentation reduces antinutritional factors in mulberry leaves while increasing probiotics and bioactive compounds, and that 10% FML improved nutrient digestibility, intestinal morphology, and antioxidant status in finishing pigs. Jiayu et al. (23) found that 2% mulberry leaf powder did not affect growth performance in weaned piglets, whereas 6% had negative effects. The absence of adverse effects at 20% FML in this study may be attributed to the bacterial–enzymatic co-fermentation, which effectively reduced tannins and fiber while releasing bioactive compounds such as flavonoids (39), polysaccharides, and alkaloids known to support growth and immunity (40). The 60-day fermentation period likely contributed to the successful degradation of crude fiber, enabling higher inclusion levels without compromising performance.

As FML inclusion increased, apparent digestibility of DM and CP decreased significantly, consistent with findings by Jiayu et al. (24). Although fermentation reduced fiber content, the neutral detergent fiber level in FML was 42.60%, and dietary fiber increased substantially at inclusion levels ≥ 10%. Dietary fiber can elevate digesta viscosity, hindering enzyme–substrate interaction and reducing DM and CP digestibility (41). Conversely, EE digestibility increased significantly in the 15% and 20% groups, possibly due to bioactive substances released during fermentation, such as flavonoids and small peptides, which may emulsify fats or activate lipase (42). Lower intestinal pH (Table 5) may also support lipase activity (43). Peng et al. (38) similarly observed increased EE digestibility with 10% FML in finishing pigs. Although DM and CP digestibility declined, growth performance remained unaffected, possibly because improved gut health and reduced diarrhea offset nutrient losses.

Intestinal pH is a key determinant of microbial activity and nutrient digestion (44). Acidic conditions inhibit pathogens while promoting beneficial bacteria such as *Lactobacillus* and *Bifidobacterium* (45). In this study, duodenal pH was significantly lower in all FML groups than in the control group, and ileal and cecal pH also tended to decrease with increasing supplementation levels, which was highly consistent with the observed reduction in diarrhea rates. The 15% group showed a 52.46% reduction in diarrhea rate during the 28–42 day period and a 41.86% reduction over the entire period, representing the most pronounced effect. Yan et al. (46) found that mulberry leaves could reduce the incidence of diarrhea by increasing villus height, improving intestinal integrity, enhancing tight junction expression, improving intestinal barrier function and increasing antioxidant capacity. Mulberry leaf extract has been shown to increase the numbers of *Bifidobacterium* and *Lactobacillus* in the intestine of weaned piglets, reduce the number of pathogenic *Escherichia coli*, lower colonic pH, and alter colonic microbial fermentation patterns by increasing the concentration of SCFAs derived from carbohydrate fermentation (47). In this study, the numbers of *Lactobacillus* and *Bifidobacterium* in the jejunum and cecum were generally higher in the FML groups than in the control group (Table 5), further confirming that the improvement in intestinal pH and diarrhea reduction by FML is closely associated with the promotion of beneficial bacterial proliferation.

Short-chain fatty acids (SCFAs) are organic linear carboxylic acids with fewer than six carbon atoms, mainly produced by the fermentation of dietary fiber by intestinal microorganisms, with acetate, propionate and butyrate being the most abundant (48). Acetate is primarily produced by *Bifidobacterium, Bacteroides* and *Ruminococcus*, and can lower intestinal pH, exert anti-inflammatory effects, influence fat deposition and enhance intestinal epithelial barrier function (49). Propionate serves as a precursor for gluconeogenesis, improving insulin sensitivity, reducing fat synthesis, lowering serum cholesterol and regulating the gut microbiota to strengthen the intestinal barrier (50). In this study, the cecal acetate and propionate contents in the 10%, 15% and 20% FML groups were significantly higher than those in the control group, with increases of 38.98%−47.05% for acetate and 45.07%−59.33% for propionate, while butyrate content showed no significant change. These findings are consistent with the results of Cui et al. (25) in finishing pigs, indicating that FML can promote the production of acetate and propionate. No significant differences in butyrate were observed. This may be due to the sampling site (cecum) and warrants further investigation across the entire hindgut. The increase in acetate may be related to the utilization of fiber components in FML by microorganisms, and the large number of lactic acid bacteria present in the FML themselves may synergize with the indigenous gut microbiota to promote acetate production. The increase in propionate is closely associated with the increased relative abundance of propionate-producing bacteria such as *Prevotella*. The increase in SCFAs not only explains the decrease in intestinal pH but also provides a metabolic basis for the reduction in diarrhea—SCFAs have anti-inflammatory and intestinal barrier-enhancing properties.

Gut microbiota research in animals and humans' advances, revealing diverse functions (51, 52). Increasing evidence underscores the pivotal function of intestinal microflora in modulating intestinal inflammation and barrier integrity (53, 54). The effects of FML on the intestinal microbiota of weaned piglets were systematically analyzed using a combination of traditional culture-based enumeration and 16S rRNA sequencing. The culture-based results showed that FML promoted the proliferation of *Lactobacillus* and *Bifidobacterium* in the jejunum and cecum, which was consistent with the sequencing results. α-diversity analysis revealed that supplementation with FML reduced bacterial dominance (as indicated by the decreased Simpson index) and increased phylogenetic diversity (as indicated by the increased PD index), making the microbiota more even. β-diversity analysis showed that the microbial structure of the 15% group was distinctly different from that of the other groups, which aligned with the trends in diarrhea rate, SCFA content and other indicators, further supporting that 15% was the optimal supplementation level.

A caveat regarding the 16S rRNA sequencing is the small sample size (*n* = 3 per group). While this sample size is common in exploratory microbiome studies, we acknowledge that it may not fully capture the within-group diversity. Future studies with larger sample sizes are needed to confirm these findings. Additionally, the live lactic acid bacteria present in FML (1.05 × 10^11^ CFU/g) may have directly colonized the piglet gut and contributed to the observed changes. We cannot completely separate the effects of live bacterial transfer from those of fermentation-derived metabolites. Future research using heat-inactivated FML or sterile filtration would help address this question. Another limitation of this study is the absence of a positive control group (e.g., an antibiotic or a probiotic). Without such a comparison, the relative efficacy of FML against established anti-diarrheal treatments cannot be determined. Future studies should include a positive control to benchmark the effects of FML.

At the phylum level, the dominant phyla were Firmicutes and Bacteroidetes, consistent with the typical intestinal microbiota composition of pigs. At the genus level, *Prevotella* was the most abundant genus, with the highest relative abundance in the 15% group (22.46%). *Prevotella* is a common fiber-degrading bacterium in the pig intestine, capable of breaking down plant cell walls to produce SCFAs, and its abundance is associated with high-fiber diets (55). In this study, the highest relative abundance of *Prevotella* in the 15% group corresponded to the increases in acetate and propionate, indicating that FML promoted SCFA production by enriching fiber-degrading bacteria. The relative abundance of *Lactobacillus* was higher in all supplementation groups than in the control group, which is consistent with the culture-based enumeration results. The relative abundance of *Bifidobacterium* showed an increasing trend in the 10% and 15% groups, similar to the findings of Cui et al. (25), who reported that FML increased the abundance of *Bifidobacterium* in the colon of pigs. *Bifidobacterium* can produce lactate and acetate, stimulate the release of anti-inflammatory factors, participate in intestinal inflammation repair, and protect host intestinal health by competitively inhibiting the growth of harmful bacteria (56).

Kruskal–Wallis tests revealed that the relative abundances of seven genera differed significantly among groups. *Prevotella* was most abundant in the 15% group, significantly higher than in the control and 5% groups. *Lactobacillus* was most abundant in the 10% group, significantly higher than in the control group. *Bifidobacterium* was most abundant in the 15% group, significantly higher than in the control and 20% groups. *Lachnospiraceae_NK4A136_group* was most abundant in the 20% group, significantly higher than in the control group. These differentially abundant genera are primarily SCFA-producing bacteria, consistent with the observed increases in SCFA content. In contrast, the control group had a higher abundance of some unclassified potential opportunistic pathogens, further confirming that FML optimizes the intestinal microbiota. This is consistent with the mechanism by which natural active components in mulberry leaves (such as flavonoids and polysaccharides) regulate microbial structure and inhibit harmful bacteria (41). Studies have shown that mulberry leaf polysaccharides can inhibit *E. coli* and promote the proliferation of *Lactobacillus* and *Bifidobacterium* (57), and that mulberry leaf powder can indirectly regulate the gut microbiota and improve the epithelial barrier through the Keap1–Nrf2 signaling pathway by modulating tight junction protein expression and reactive oxygen species production (23).

## 5 Conclusions

Dietary inclusion of 5–20% FML did not significantly influence growth performance in weaned piglets, although the 20% level tended to increase ADG and reduce F/G during days 42–56. Supplementation at ≥10% significantly reduced DM and CP digestibility but increased EE digestibility. The 15% FML group was the most effective in reducing diarrhea (41.86% overall), lowering duodenal pH, and increasing cecal acetate and propionate levels. FML supplementation also altered cecal microbial composition, reducing bacterial dominance and enriching beneficial genera such as *Prevotella, Lactobacillus*, and *Bifidobacterium*. Collectively, 15% FML emerged as the optimal level for improving intestinal health and reducing diarrhea without compromising growth performance, through mechanisms involving modulation of gut microbiota, enhanced SCFA production, and reduced intestinal pH. Practical limitations of using 15% FML in production include the cost of raw mulberry leaves, batch-to-batch variability in fermentation quality, and the need for a stable, year-round supply. Future research should include cost-benefit analyses and large-scale validation trials before recommending widespread adoption.

## Data Availability

The 16S rRNA amplicon sequences have been deposited in the National Center for Biotechnology Information (NCBI) Sequence Read Archive (SRA) (https://www.ncbi.nlm.nih.gov/sra/?term=PRJNA1444798), under accession number PRJNA1444798.
